# On Tubercles of the Testis

**Published:** 1840-04-25

**Authors:** Henry H. Smith

**Affiliations:** Paris


### MEDICAL EXAMINER.

DEVOTED TO MEDICINE, SURGERY, AND THE COLLATERAL SCIENCES.

No. 17.] PHILADELPHIA, SATURDAY, APRIL 25, 1840. [Vol. III.

### ON TUBERCLES OF THE TESTIS.

BY HENRY H. SMITH, M. D.

The subject of tubercles, which has so long been regarded as peculiar to the province of the physician, and which, from their frequent occurrence, forms so important a portion of the diseases daily brought to his notice, promises, from the facts constantly adduced by the industry of the French pathologists, to prove of no less interest to the surgeon. The excellent work of Mons. Nelaton on these bodies in the bones, the treatises of Mons. Guerin on the same productions in causing curvature of the spine, and the observations incessantly made on their course in the testicle, clearly prove that they play no unimportant part in the affections confided to surgery. Though tubercles, when developed in the testis, do not require the same constant attention as when appearing in more vital organs, yet they demand, from the influence which they exercise on the mind and happiness of the individual, as well as from the unpleasant symptoms accompanying their progress, to be specially regarded by any one not content with a mere routine practice of his profession. Under the title of Scrofulous Inflammation of the Testis, Sir Astley Cooper has referred to this disease in his splendid work on the Testicle; but the vagueness of the term he has given, and the indefinite nature of its cause, may, it is thought, be sufficient to justify a change in this respect, and the adoption of the more scientific one employed by Mons. Berard, jr., and placed at the head of this paper. From the position occupied by Mons. Berard, in a hospital devoted to the affections of the generative organs, (Chirurgien à l'hôpital des Veneriens à Paris) he has had many opportunities of investigating this subject, and in the compilation of this paper I have availed myself of his excellent article, as well as of the splendid work of Mons. Cruveilhier.

In the testicle, as in other organs of the economy, tubercles present themselves under two forms, either as isolated masses, forming the disseminated tubercles, or as infiltrated, and generally spread throughout the testis, epididymis, vasa deferentia or prostate gland. In the disseminated ones, we sometimes find only a single tubercle, which presents the characters common to these bodies, but it is more usual to see four or five existing at the same time, under the form of little tumours, more or less regularly rounded, hard, almost insensible to pressure, moveable under the scrotum, and firmly imbedded in the portion where they have been discovered, and of a size varying from a buckshot to a cherry stone, or even a chestnut. When small, they lodge in the cellular substance between the vesiculæ seminiferæ without appearing to affect these tubes; but when of greater size, they invade the filaments of the testicle and destroy more or less of its substance. The infiltrated tubercles generally occupy the body of the testis, taking the place of its true substance, or when deposited in the epididymis, completely change the appearance of the part, presenting to the touch masses of different sizes, with a hardness altogether different from the usual elastic feel of the tube. In some instances, the epididymis is enlarged to triple its ordinary size, and seems to be completely injected with the tuberculous matter in a semi-liquid state. This disease rarely confines itself to one testicle, though it is rare to see both in the same state of development; but whether this arises from their proximity or from both being exposed to the same exciting cause, it is difficult to say. In a case seen by Mons. Berard, the left testicle, which was in the scrotum, had been attacked with the disease for two years, whilst the right, which had remained in the inguinal canal, had only commenced to suffer six weeks previous to his seeing it. When the epididymis is the portion affected, the tubercles commence most frequently in the globus major, and, in a majority of the cases, will be found to be formed by the isolated tubercles, and to be of easy diagnosis.

Tubercles of the testis rarely attack children, at least till the years near to the period of puberty, as these organs are generally too little developed previous to this time, to admit of their deposition. In adults, they are found generally between the ages of 16 and 40, attack, in preference, those of a lymphatic temperament, or in whom we might suspect tubercles elsewhere, and frequently follow the suppression of a gonorrhœa. Many of the cases reported by Mons. Cruveilhier had had syphilis without gonorrhœa, others had received a contusion, others had suffered from repeated gonorrhœas, the last of which, however, had generally preceded by some months, the appearance of the disease, and in others, the causes were unknown. In the few cases I have seen, the disease could generally be referred to the constitution of the patient, as a predisposing cause, though, in every instance, the exciting one was an external injury received in riding, climbing, &c., or from the rudeness and want of care in the treatment of a gonorrhœa. In one case, the commencement could be traced to a blow given by the fist of the patient whilst in a paroxysm of anger at the duration of a chordee, which he thought to conquer in this manner.

The changes occurring in the development of the tubercles are seldom perceived by the patient at an early period, as the tumefaction of the scrotum commences without pain or change in the colour of the skin, increases slowly, and can exist for months without other inconvenience than that resulting from the extra size of the part, and it is not until the tubercles are numerous, or reach an advanced state, that the signs of their existence become sensible to him. At this time the scrotum presents, frequently, a thickness and size double that which it has in the healthy state, and the tubercles present to the touch a well rounded form, with some little iregularities, also more or less rounded, and of the firm texture so well recognised when felt in the lungs. But after a certain time, which varies considerably, but is always tedious, the tubercles commence to soften and to pursue the changes so well known from the works of Bayle and Laennec; the scrotum, immediately over the tubercle, becomes of a red colour, more or less livid, sometimes even bluish, and thinner and thinner till it terminates in ulceration, when the tubercle escapes in a yellowish pus-like liquid, generally furnished at the expense of the cellular substance, in which it is easy to recognise the caseous particles formed by the tubercle. If, instead of permitting the ulceration of the skin, and the escape of the tubercle by suppuration, it is cut into soon after its formation, the matter will be found in a concrete form, and will escape, of itself, through the artificial opening, by the simple contraction of the surrounding parts. The course of these bodies to suppuration is not the same in all cases, as regards the duration; and in the same case, one nearly always progresses more rapidly than another, so that it is not uncommon to see in the same testicle, one abscess opened and discharged, and find newly developed tubercles still in a hard state within a few lines of it. When seated in the epididymis, their course to a discharge externally is always more rapid than when situated in the body of the testicle, the fibrous coat of the latter always opposing, strongly, a suppuration towards the surface. After the discharge of the matter, the scrotum presents an appearance highly characteristic of the disease. At this time we find one or more cavities or depressions, externally of an unequal surface, communicating with the cavities internally by a course more or less direct, but most frequently tortuous and fistulous. The internal cavities are points caused by the inflammation consequent on the suppuration of the tubercle, and are divided sometimes into little cells, by irregular partitions, the sides of which are filled with softened tuberculous matter, which preserves the discharge for a long period. As these close, a termination much to be desired, but very difficult to obtain, there is left an irregularly depressed cicatrix, of the size of a small shot or pea, which always shows more or less loss of substance, and marks, for ever, the fistulous opening caused by a softened tubercle. But most frequently these openings constitute the most annoying symptom in the case, as, after the tubercle is discharged, we do not find it easy to cause adhesions, owing to the moveable nature of the parts, the constant discharge and the hardness of the parietes of the fistulæ. In some cases the difficulty is increased by the discharge of the seminal fluid, especially during dreams or the venereal orgasm. After the opening has remained a few days, nature attempts the cure by the formation of granulations about the fistulæ. Should the case be presented to us at this moment, it would not be difficult, at first sight, to mistake it for an ulcerated cancer of the testicle, from the tint of the skin, the increased size and hardness of the parts, and the fungous granulations; but a little reflection will solve the difficulty, and render clear the diagnosis.

Tubercles are never the seat of lancinating pains; their compression does not cause pain, at least till the surrounding tissue is inflamed; their hardness is less than that of scirrhus, and greater than that of the encephaloid tissue; they are almost always numerous, and developed in the epididymis in preference to the testicle. Cancer, on the contrary, prefers the body of the testis. Tubercles go through peculiar well marked changes from deposition to softening, their surface is smoothly rounded and circumscribed to the touch; scirrhus on the contrary is generally irregular, is found in lobulated masses of an irregular shape, and seldom in its commencement prevents our feeling the healthy portions of the testis at a point *distinct* from its seat. Tubercles most frequently are developed indiscriminately, and if we feel the testis, it is in the intervals *between* each tumour, and when they reach the period of softening, their course is indolent, the discharge is peculiar, the lymphatic ganglions undergo no change, and the general health is not seriously affected. When on the other hand cancer ulcerates, its course is rapid, there is great sensibility even in the granulations, and the discharge is often coloured with blood. In tubercles the matter is like pus, contains portions of the tubercle in a majority of the cases, and never is coloured with blood, unless produced by accidental causes.

Should the substance of the testicle have not been affected, we can generally distinguish its usual size and figure, and its functions will not be sensibly changed. But should the tubercles originate in its body, the affection is much more serious, as the seminiferous ducts can escape from the openings, as in the cases reported by Swediaur* and a consequent weakness or atrophy be produced. Dupuytren has remarked that in some cases where the disease existed a long time, "the testicle became softened, and fungous, and similar to the tissue found around the articulations attacked with white swelling."†

Such is the usual course of the isolated tubercles to a cure by suppuration, and the question naturally presents itself, as to whether there are cases which can happily terminate by resolution? Many surgeons deny entirely the possibility of such a termination, but Mons. Berard affirms that it can. Delpech also states, that he has seen cases "in which tubercles already softened have caused ulcerations, whilst new tumours, presenting the same characters and appearance as to the former at a like period, have disappeared completely, either under a proper treatment or by the increased action in the parts consequent on sexual intercourse or the changes of puberty." He further says, "that there are even facts to prove that this true resolution of subcutaneous tubercles, can be favoured or decided by the continued action of cantharides over the part corresponding to the organic lesion."‡

In the infiltrated tubercles, the case is more serious and more difficult of diagnosis, as the matter is very generally deposited throughout the whole body of the testis and epididymis. It differs, however, from the isolated, in the changes which it produces, being more general and less sensible to the touch, as in the body of the testis the matter is developed in its very substance, radiating the whole length of the fibrous prolongations of the corpus highmorianum, and penetrating to the centre. In this state the whole body of the testis enlarges gradually, has a variable hardness, according as the matter is near or far from the surface, and an insensibility to pressure entirely different from the natural state, little or no pain being produced by firm compression when the matter is in a crude state, and has filled a large portion of the testicle. When it is in the epididymis, we find the whole or large portions of it engorged and knotted, affording to the touch the sensation of their being filled with caseous matter in which some portions remain harder than others. In a case of infiltration of tuberculous matter in the epididymis, seen by Mons. Cruveilhier, the tunica albuginea was thickened so as to separate it from the body of the testis, the former of which was one tuberculous mass, so much enlarged, that it was almost impossible to find any sign of the primitive tissue, and the matter had softened in spots, the largest of which was in the globus major. In the isolated tubercles, dissection generally reveals a portion of the structure untouched by the disease, but on the whole the diagnosis is difficult between these and the infiltrated, the great difference being, that the isolated form numerous globular tumours, *distinct* and saliant at the surface, whilst in infiltration there is a general increase of the part in size and density without there being much alteration in form.

Tuberculous infiltration frequently destroys a part or the whole of the functions of the organ, and its cure is always difficult. As a tuberculous affection, two questions of considerable interest present themselves.

1st. Has the patient affected with tubercles of the testis, necessarily the same bodies developed in the lungs?

When we consider that tubercles of the testis, most frequently affect persons of a lymphatic temperament, and that they are developed at an age when phthisis pulmonalis is most common, we might reasonably fear that this complication would exist, at least in a great number of cases. Mons. Berard, however, cites but few where the disease invaded the lungs and testicle at the same time; in all the others, the testicle alone appeared to be affected, and the patient offered no sign of a pulmonary affection, even some time after their cure.

2d. Ought the presence of tubercles in these organs, to authorize the operation of castration, in order to prevent their development in the more important parts?

As we know nothing to prove that tuberculous matter when absorbed has the power of provoking the formation of these tumours in other organs, we might readily answer in the negative. Mons. Cruveilhier, however, regards this question, as depending on *the seat* of the tubercles; and says, that when the tuberculous matter exists in the epididymis, we ought not to have recourse to castration, but that when it is in the body of the testis, the operation may be necessary to relieve the existing symptoms. Yet, as castration is often fatal in its results, he advises the attempt to cure the affection, even if obtained at the expense of an atrophied testicle, and the consumption of a long period in the treatment. Mons. Berard also opposes decidedly the operation of extirpation, preferring the cutting into and removal of the portion affected, to the entire removal of the gland. It ought, however, to be noted as an observation on the part of Mons. Cruveilhier, that where the *tuberculous* testis has been removed, the success has been nearly constant and without a reappearance of the disease in other parts; whereas the reverse is the case in the extirpation practised for cancer, the success being much less, and the relapses much more frequent.

Can the tuberculous testis pass readily to a cancerous state?

The opinions on this point seem to be very much divided, many believing that it can.

* Dictionnaire des Sciences Medicales.

† Lecons orales.

‡ Maladies Chirurgicales, tome III, pages 633 and 635.

Nevertheless Mons. Berard, denies it entirely, and says, that in cases where the cancer has appeared to succeed tubercles, there has existed a complication of the two diseases. Mons. Cruveilhier likewise reports one or two cases of a similar complication, and one case under my own observation in the wards of Mons. Velpeau, at La Charité, supported the same opinion.

*Salle St. Augustin.*

*No.* 38.—Pimber, cabinet-maker, aged 36 years, entered February 19th, 1840, with an enlargement of the right testis. The testicle was of the size of an egg, oblong, irregular in shape, being larger at its upper portion, lobulated, and presenting different degrees of con sistence. At the lowest part it is elastic, soft, painful on pressure, and evidently belongs to a sound part of the testicle; above this on the inner portion towards the raphé, is a small fluctuating point, external to which is a harder portion, connected apparently with the firm lobulated structure, forming the enlargement at the upper extremity. The epididymis is readily felt at the lower part, in a healthy state, but at the upper is not to be distinguished from the mass of the testis; the cord is natural, the scrotum is soft, relaxed, moveable over the tumour, and exhibits two cicatrices, from which the patient says matter escaped nearly eight months ago. General health excellent, complexion good, has had both chancres and gonorrhœa, but was never mercurialised; he dates the disease back to 1838, two years since; does not recollect how it commenced, but believes the cause to have been a blow received in mounting a horse; the scrotum he states was always long, and wearing loose pantaloons, the parts were caught under him as he sprung from the ground to the horse's back. He was in the hospital eighteen months since, but left it, being unwilling to submit to treatment. Since that period has undergone every remedy, as leeches, scarification, hydriod. potass. iodide of mercury, fumigations, &c., but without any change in the tumour, which has continued to increase, and progressed rapidly within eight or ten weeks. The operation of castration was performed on him, by Mons. Velpeau, February 24th, 1840, by means of an incision in the scrotum, turning out the testicle and cord, the latter of which, with all its vessels, &c., was included in a ligature and divided by the bistoury. On opening the testicle longitudinally, several strictures were presented. The upper portion was composed of an encephaloid tissue which occupies nearly the whole of the shell of the testis; near the middle was one large tuberculous mass, the size and shape of a chestnut, with one or two smaller ones near it. The cyst contained a fluid not recognised as peculiar to any of these portions, and the lower part of the testis was unaltered, with the exception of a small tubercle near its centre; the epididymis was sound throughout. The wound was healed by granulation, and the patient is now, March 17th, nearly recovered, having had no bad symptoms. The other testicle is perfectly healthy.

*Treatment.*—The constitutional means likely to counteract the lymphatic temperament of the individual, constitute the most important part of the treatment during the whole course of the disease, such as the use of mild tonics, especially the ferruginous preparations, and all those remedies which the knowledge of the predisposing cause of the disease would indicate. The avoidance of all means likely to produce a contusion of the scrotum, or an inflammation of the parts, ought also, it is hardly necessary to say, to be strictly enjoined. The local remedies will vary according to the state of development of the tubercles; thus, during their crude state, the employment of means likely to produce their resolution, as irritating friction, which, by increasing the circulation, may change the vitality of the part, as preparations of iodine, of mercury, or of cantharides, continued for some time, and afterwards covering the parts with a soap plaster and the constant use of a suspensory truss. Nevertheless, we must watch carefully the action of these substances, lest the inflammation should go too far, and produce a suppuration of the tubercle, a termination always to be avoided. The complaint of pain on the part of the patient, ought, therefore, to be the signal for the use of emollients. But in most cases it will be in vain to attempt a resolution, as the natural tendency of tubercles here, as of other foreign bodies, is to an escape by suppuration, the tubercle not only producing an inflammation of the parts around it, but this inflammation causing suppuration, which will sooner or later terminate in ulceration.

When the matter is once formed, ought we to wait till fluctuation be perfectly distinct before opening it, or ought we rather to give a prompt issue to the softened portions ?

Mons. Berard, advises strongly an early opening, as tending to prevent any considerable thinning of the skin, and the formation of fistulæ or even a true abscess between the softened tubercle and the scrotum. The effect of allowing the natural discharge of this matter by ulceration can not be better shown than by the following case.

*Salle St. Ferdinand.*

*No.* 10.—Paquier, aged 41 years, carter, entered the wards of Mons. Velpeau, on the 28th of January, for an affection of the testicle. In May, 1839, whilst loading his cart, he received a blow on the testicle, from a bar of iron, which produced the pain and faintness usual immediately after an injury to this organ. It did not, however, prevent his continuing his work till towards September, when the swelling and hardness of the parts induced him to consult a physician, by whom he was leeched, kept in bed, and had ointments rubbed on the scrotum. After a continuance of this treatment, he was again obliged to resume his work, and continued at it without noticing particularly the parts, till the 8th of November, 1839, when it became painful and inflamed externally. This obliged him again to consult his physician, by whom he was leeched and afterwards poulticed, but was obliged to continue at his work till the end of December, when he was confined to his bed, and again submitted to treatment of a similar nature till an abscess opened in the lower part of the scrotum, when he was sent to the hospital from want of funds. On his entrance, the left testis was the size of a large egg, the scrotum was very much relaxed, of the natural colour, at the upper part, but below was of a deep bluish red, especially at the sides of the abscess, where the skin was very thin, ragged, and disposed to sloughing. The cavity made by the abscess would admit the end of the fore-finger, and the matter had extended under the integuments of the perineum, where a second abscess had formed. This was freely opened by Mons. Velpeau, and gave exit to a pus like matter, mixed with a little blood. A further examination of the testicle, showed the epididymis, to contain tubercles throughout its whole extent, presenting hardened masses, which I cannot better describe than by saying as if filled with peas, some of which had been mashed. The testicle also was enlarged, but seemed to be more the result of the inflammation near it than of disease in its substance, its sensibility and elasticity being preserved. The right testicle is perfectly natural, as is also the scrotum on this side of the raphé, but several tubercles in a crude state can be felt in the upper part of the epididymis, of which the patient is not aware, as he complains of nothing unnatural in this gland. His general health has always been good, and he has never before had an affection of the testicle, though he suffered from chancres when young.

After the opening of the abscess, Mons. Velpeau directed the use of cataplasms to the part, and good diet, under which treatment he continued for some time; the edges of the scrotum having sloughed off, and showed a tendency to granulation, when a change took place, the ulceration seemed to have recommenced, and he has had several hæmorrhages from the part, without it being possible to distinguish the point from whence it comes. He is now, March 26th, 1840, still under treatment, with the prospect of having to submit to castration.

It hardly admits of a doubt that had this case been differently treated, previous to its entrance into the hospital, much of these results could have been prevented, as a free incision would have prevented the burrowing of the matter, the ulceration of the skin, and left the parts in a state more favourable to cicatrization. If, however, fistulæ are formed by the natural discharge of the matter, we must after giving free vent to the discharge, attempt to provoke adhesion by stimulating injections, of which, those of port wine, lime water, a solution of sulphate of copper, of the nitras argenti, or of tinct. cantharid. are the most constantly employed. Should the fistulæ still continue, the treatment must be persevered in, varying the injections, or adding thereto, as recommended by Mons. Berard, douches of sulphurous, saline, saponaceous or chalybeate waters. The removal of the hardened sides of the fistulæ by the knife, or by caustics, as the acide nitrique de mercure, will frequently hasten their adhesion. Notwithstanding, sometimes, the employment of all these means successively, the duration of the fistulæ will be tedious, but they will heal sometimes of themselves, though after a long period. In the case of a sailor, who entered La Charité, in October, 1839, for a contraction of the tendons of his feet, numerous tubercles of the testis in a crude state were found in the right testicle, whilst several cicatrices remained in the scrotum, from which, according to his account, matter resembling shreds of beef had escaped for a long time. He stated that when in Africa, nearly three years previous, he had bruised his testicles by falling on some rocks, but had never been treated for it. Some months after, matter was discharged from the scrotum, without his suffering very acute pain, and this matter continued to escape by different openings from time to time, during two years, when the fistulæ closed without treatment, and he thought so little of their existence, that he had said nothing of it on his entrance to the hospital.

The great difficulty of obtaining the resolution of tubercles, makes us foresee that their softening will bring on ulcerations in the scrotum, and fistulous openings, difficult to cure. Would it not, therefore, be better to anticipate this termination, and incise at an early period the hardened and encysted tubercle? Would not also the wounds resulting from this operation be more disposed to a prompt cicatrization? In one case, where the brother of Mons. Berard recognised the presence of large tubercles in the testicle, a large incision made on the part caused the exit of the mass by means similar to the extraction of a kernel from a nut, and the cure was radical and prompt: but the testicle rested completely atrophied. Perhaps it would have been possible to have prevented this atrophy by incising the tubercle alone, avoiding any injury to the substance of the testis, and in the epididymis it would most probably be always successful. It would, however, be only applicable to cases, where the tubercles were isolated, when, as has been shown, the seminiferous tubes are rarely affected by their existence.

*Paris, March 27th*, 1840.

*Pennsylvania Hospital, April* 29, 1840.

To the Editors of the Medical Examiner.

*Gentlemen,*—Agreeably to the request of Dr. J. F. Meigs, previous to his departure for Europe, I am happy to inform you, that J. C——, the case reported in No. 13 of the present volume of your periodical, for compound fracture of the thigh with some loss of bone, was this day discharged, *cured.*

Respectfully, yours, &c.

ANTHONY E. STOCKER.

